# Reduced Oxygen Extraction Fraction as a Biomarker for Cognitive Deficits in Obstructive Sleep Apnea

**DOI:** 10.1002/brb3.70273

**Published:** 2025-02-06

**Authors:** Mahdi Mohammadi, Shahram Samadi, Seyed Amir Hossein Batouli, Khalil Pestei, Mohammad Ali Oghabian

**Affiliations:** ^1^ Department of Medical Physics and Biomedical Engineering, School of Medicine Tehran University of Medical Sciences Tehran Iran; ^2^ Neuroimaging and Analysis Group, Research Center for Molecular and Cellular Imaging, Advanced Medical Technologies and Equipment Institute Tehran University of Medical Sciences Tehran Iran; ^3^ Sleep Breathing Disorders Research Center, Imam Khomeini Hospital Complex, School of Medicine Tehran University of Medical Sciences Tehran Iran; ^4^ Anesthesia, Critical Care and Pain Management Research Center Tehran University of Medical Sciences Tehran Iran; ^5^ Department of Neuroscience and Addiction Studies, School of Advanced Technologies in Medicine Tehran University of Medical Sciences Tehran Iran; ^6^ Pain Research Center, Neuroscience Institute, Anesthesiology Department, School of Medicine Tehran University of Medical Sciences Tehran Iran

**Keywords:** Cognition, MRI, Obstructive sleep apnea, Oxygen extraction fraction, Quantitative susceptibility mapping

## Abstract

**Background:**

Obstructive sleep apnea (OSA) is characterized by disruptive breathing, resulting in a decline in cognitive performance. This study investigates the role of oxygen extraction fraction (OEF) and quantitative susceptibility mapping (QSM) in OSA‐related cognitive impairment.

**Methods:**

The study recruited 15 patients with confirmed OSA and 16 healthy controls, who underwent overnight polysomnography and brain MRI using a 3 Tesla machine and 64‐channel head coil. A two‐step MRI analysis was employed to measure OEF. QSM was first created by processing separate phase and magnitude images. OEF maps were then generated by identifying veins based on their susceptibility. Volumetric analysis was performed using the FreeSurfer. Neuropsychological tests were administered to evaluate cognition.

**Results:**

The analysis of OEF revealed significantly lower values in various cerebral cortical regions of OSA patients than in controls. Notably, OEF in the cerebral cortex and frontal, temporal, and occipital regions showed negative correlations with the duration of stage N2 sleep (highest correlation between N2 and right temporal OEF: *p* = 0.005, *r* = −0.681). Furthermore, poorer performance on neuropsychological tests, such as the backward digit span test, was significantly correlated with reduced OEF in the left hemisphere (*p* = 0.016), left cerebral cortex (*p* = 0.019), right frontal (*p* = 0.034), left frontal (*p* = 0.014), left parietal (*p* = 0.008), left temporal (*p* = 0.048), and left occipital lobes (*p* = 0.015). No significant differences in QSM or brain volume were observed.

**Conclusions:**

Decreased OEF emerges as a potential biomarker for cognitive deficits in OSA, suggesting disturbances in cerebral oxygen metabolism may underlie cognitive impairments. These findings underscore the importance of investigating physiological markers in understanding OSA‐related cognitive dysfunction.

## Graphical abstract

The OEF map was calculated and generated by QSM. The analysis of OEF revealed significantly lower values in various cerebral cortical regions of OSA patients than in controls. Decreased OEF emerges as a potential biomarker for cognitive deficits in OSA, suggesting disturbances in cerebral oxygen metabolism may underlie cognitive impairments.

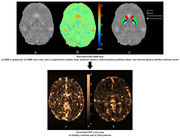

## 1 Introduction

Sleep‐related breathing disorder is characterized by abnormal respiration during sleep. The most common disorder in this category is obstructive sleep apnea (OSA), which is defined by frequent episodes of partial or complete upper airway closure, resulting in disruptive breathing during sleep (Najafi et al. 2021). OSA affects a significant portion of the general adult population worldwide, with estimates ranging from 9% to 38% based on a systematic review, and is notably higher in men than in women (Senaratna et al. 2017). It correlates with reduced quality of life and multiple health issues that affect the cardiovascular and central nervous system (Ghaderi et al. 2023).

Microarousal, sleep fragmentation, intermittent hypoxemia, and hypercapnia are driven by repetitive apneas and hypopneas. These conditions can also result in increased sympathetic activity, oxygen sensor activation, oxidative stress, and chronic systemic inflammation. Ultimately, this may lead to changes in the structure and function of the brain associated with OSA, as well as deficits in memory, executive function, and attention (Park et al. 2022).

Many studies have utilized structural and functional neuroimaging to discover the connection between changes in different brain regions and cognitive performance (S. Mohammadi, Mohammadi, and Ghaderi 2023; M. Mohammadi et al. 2024; Chen et al. 2018). However, while some studies may enhance our understanding of cognitive functions and alterations in imaging parameters (Hashim et al. 2024), the precise neuronal mechanisms by which OSA contributes to cognitive impairment, especially related to structural and functional imaging, remain unclear. Iron plays a crucial role in various biological processes in the brain, including oxygen transport and neurotransmitter metabolism, and while proper iron homeostasis is essential for normal brain function, its misregulation can lead to neurotoxicity and oxidative damage (Wang and Pantopoulos 2011; Ward et al. 2014). Excessive iron levels can worsen the oxidative stress and inflammation caused by OSA, leading to brain cell damage and subsequent cognitive alterations (Liu et al. 2020). In addition, impaired oxygen delivery and utilization can potentially change the cerebral oxygen extraction fraction (OEF) in patients with OSA.

OEF is an essential physiological measure of brain energy metabolism and cerebral oxygen usage (Jiang and Lu 2022). The information offered regarding proportional deficits in cerebral blood flow in relation to oxygen consumption throughout the tissue (Miyata et al. 2019). Many approaches, including blood oxygen level‐dependent (BOLD) and quantitative BOLD (qBOLD) methodologies, have been developed to noninvasively estimate OEF without utilizing ionizing radiation owing to advancements in magnetic resonance imaging (MRI) techniques. To measure OEF, these methods model the relationship between tissue oxygenation and magnetic susceptibility effects of deoxyhemoglobin in venules (Engle et al. 2024). However, their accuracy and repeatability are limited because they require assumptions regarding the vessel geometry and hematocrit levels (Zhang et al. 2015).

Quantitative susceptibility mapping (QSM) is an MRI technique used to measure the magnetic characteristics of brain iron in vivo (Madden and Merenstein 2023). QSM is particularly sensitive to iron content in gray matter and myelin content in white matter, resulting in positive (paramagnetic) and negative (diamagnetic) susceptibility values (Deh et al. 2018).

QSM uses the susceptibility differences between oxy‐ and deoxyhemoglobin to quantify venous oxygen saturation and derive OEF values (Kudo et al. 2016). QSM quantifies absolute OEF using a clinically easily available single MRI scan, overcoming some of the limitations of previous MRI approaches (Miyata et al. 2019). It is insensitive to venous geometry and orientation, allowing measurements of small venous structures that prior MRI methods could not identify (Ebrahimi et al. 2021).

Based on the previous literature and our comprehensive search, there are limited studies investigating OEF in the context of cognitive impairment in patients with OSA. Therefore, this study aimed to evaluate magnetic susceptibility and OEF as potential contributors to cognitive decline in this population. We compared these variables in the OSA and control groups and investigated the correlation between their levels and polysomnography (PSG) findings.

## 2 Methods

### 2.1 Participants

This study recruited 15 untreated patients with confirmed OSA (apnea‐hypopnea index (AHI) > 5) from a recent PSG at the sleep clinic and 16 age‐matched healthy controls. Participants with pre‐existing medical conditions, brain abnormalities, cognitive decline, or substance abuse were excluded from clinical interviews. The control group underwent STOP‐BANG screening performed by a sleep specialist. The STOP‐BANG questionnaire is a widely used screening tool for OSA (Pivetta et al. 2021; Waseem et al. 2021). It consists of eight yes/no questions that assess key risk factors: snoring, tiredness, observed apneas, pressure (high blood pressure), body mass index (BMI), age, neck circumference, and gender. A higher score indicates an increased likelihood of OSA, aiding in the identification of patients who may require further evaluation.

### 2.2 Overnight PSG

All patients underwent overnight PSG using miniScreen Pro (Lowenstein Medical, Bad Ems, Germany) to assess their sleep patterns. Sleep specialists validated the PSG data using dedicated software.

Under the same conditions, each patient underwent the standard PSG procedure. Our standard PSG testing included the use of all electroencephalogram (EEG) derivations, two electrooculograms (EOGs), three electromyograms (EMGs) for chin movement, two EMGs for leg movement, two electrocardiogram (ECG) leads on the chest, body position sensors, nasal flow monitoring, a small microphone for snoring monitoring, abdominal and thoracic belts to assess the respiratory effort and differentiate OSA from central sleep apnea (CSA), and pulse oximetry.

The parameters analyzed included AHI, oxygen desaturation index (ODI), snoring index (SI), arousal index (AI), minimum and mean oxygen saturation (SpO_2_) levels, REM latency, and percentage breakdown of each sleep stage (N1, N2, N3, and REM) within the total sleep time.

### 2.3 MRI Acquisition

All participants underwent standard brain MRI protocols, which included FLAIR, T1‐weighted, and T2‐weighted imaging. This was done to confirm the absence of structural lesions using T1‐weighted three‐dimensional magnetization‐prepared rapid gradient echo (MPRAGE) MRI. The scans were conducted using a 3.0T MR scanner (Prisma, Siemens Healthcare, 2016), equipped with a 64‐channel head/neck coil, at the National Brain Mapping Laboratory (NBML, Tehran, Iran). The MRI acquisition parameters were as follows: TI, 900 ms; TR/TE = 1840/2.43 ms, flip angle, 8°; and isotropic voxel size, 1 mm^3^. In addition, the susceptibility‐weighted imaging (SWI) sequence was used to calculate the QSM. The SWI settings were set as follows: TR/TE, 41/14 ms, flip angle = 15°, slice thickness = 2 mm, voxel resolution: 0.6 × 0.6 × 2.0 mm, ETL: 4, pixel spacing: 0.625/0.625 mm, FoV read = 240 mm, FoV phase = 81.3%, matrix size = 384 × 312, slice per slab = 72, slice resolution = 100%, and PAT mode: GRAPPA. The SWI sequence used full‐flow compensation to avoid phase errors and voxel displacement. Separate phase and magnitude images were generated for processing and analysis.

### 2.4 Volumetric Analysis

Axial T1‐w volumetric images were analyzed using the FreeSurfer version 7.4.1 software (http://surfer.nmr.mgh.harvard.edu). Volumetric and surface‐based segmentation were performed by FreeSurfer using a template‐driven methodology.

Motion correction, skull stripping, intensity normalization, Talairach transformation, volumetric registration, subcortical structure segmentation, white matter and gray matter segmentation, tessellation, smoothing, inflating, spherical mapping and registration, and cortical mapping and parcellation were the steps involved in FreeSurfer image processing. Finally, various volumetric and surface‐based data were generated. The output data for each participant were carefully reviewed visually.

### 2.5 QSM Reconstruction Pipeline

**FIGURE 1:**
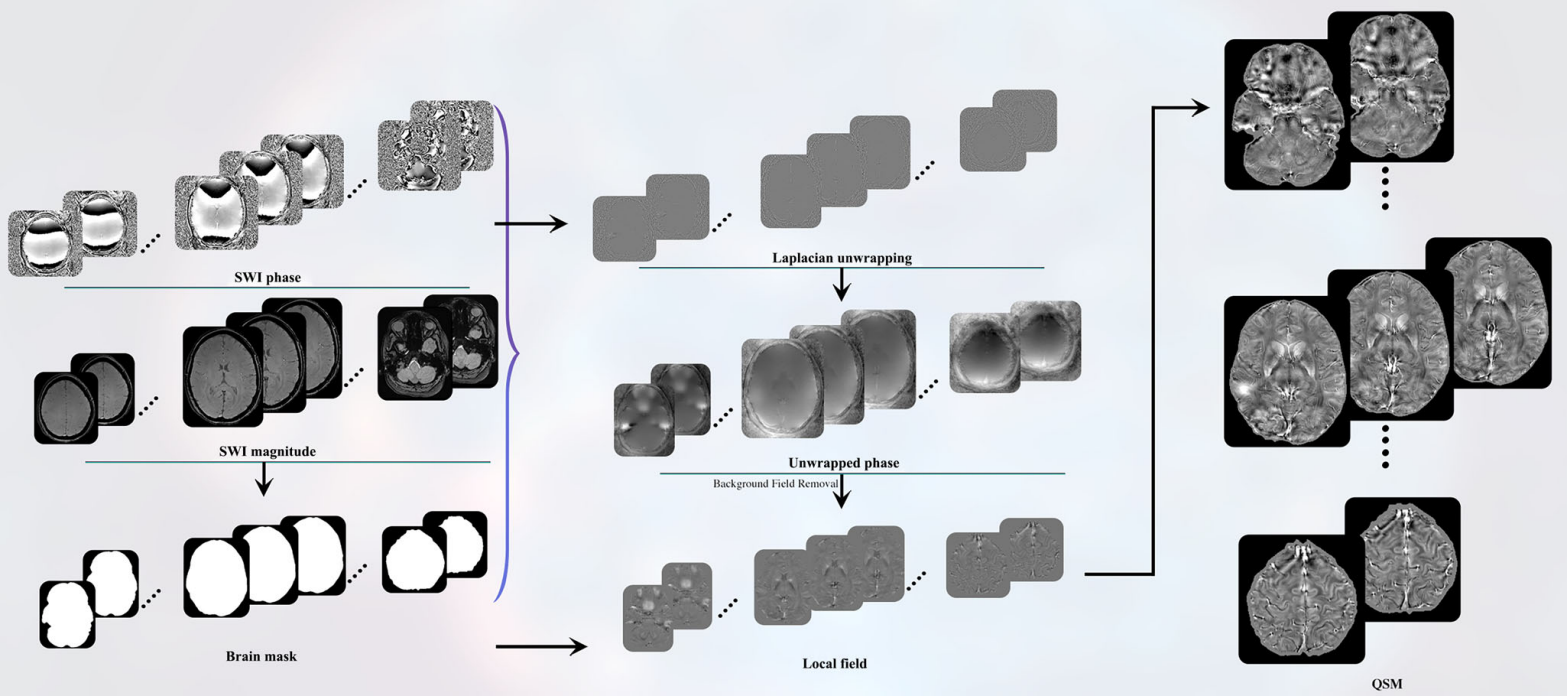
Reconstruction pipeline of QSM.

The QSM reconstruction pipeline is illustrated in Figure 1. The analysis involved preprocessing separate phase and magnitude images obtained from the SWI.

The FSL fslmaths command (https://www.fmrib.ox.ac.uk/fsl) was used to normalize the pixel values in the phase images to the −π to +π range. Subsequently, the images were unwrapped using a Laplacian‐based method within STI Suite software. STI Suite employs a Laplacian‐based approach known as phase removal, using the Laplacian operator (HARPERELLA) to unwrap 3D phases and remove harmonic background phases in a single step. HARPERELLA can efficiently remove background contributions while conserving the local tissue phase over the entire brain (Li et al. 2014). Next, a brain mask was generated from the magnitude images using the FSL brain extraction tool (BET). Background field removal and local field image generation were then performed using the following established techniques: variable kernel, sophisticated harmonic artifact reduction for phase data (VSHARP), and combining the unwrapped phase with a brain mask. To minimize boundary loss, VSHARP uses a spherical mean value (SMV) operation that progressively decreases the spherical kernel sizes over the total field. The field‐applied SMV operation with different kernel sizes is then deconvolved by the operator with the largest kernel size using the preset threshold value for truncated singular value decomposition (TSVD). To achieve a compromise between preserving the boundary and minimizing the phase error, the VSHARP approach is employed (Fang et al. 2019). Finally, the streaking artifact reduction for quantitative susceptibility mapping (STAR‐QSM) method was employed to calculate QSM. In STAR‐QSM, the dipole fields of the high‐susceptibility sources are calculated and then separated from the total phase to reduce streaking artifacts. This reconstruction method results in significantly reduced streaking artifacts and improved image quality (Wei et al. 2015).

### 2.6 OEF Map Generation Pipeline

Due to higher concentrations of deoxygenated hemoglobin, veins exhibit greater magnetic susceptibility than the surrounding brain tissue. A local threshold value of mean ± 2SD was applied within 64 × 64 × 30 defined volumes of interest (VOIs) for the whole brain to identify and isolate venous voxels. Within each VOI, the difference in susceptibility (Δ*χ*) between the veins and surrounding tissue was calculated. Established equations that incorporate Δ*χ* and hematocrit (Hct) are then used to estimate the OEF (Kudo et al. 2016). The primary equation used is as follows:

$$\mathrm{OEF}=\frac{\Delta \chi \times P_{v}}{\Delta \chi_{\mathrm{do}}\times \mathrm{Hct}}\;\left(1\right)$$

where Δ*χ* represents the susceptibility difference between veins and surrounding tissue, Δ*χ*_do_ is a constant value representing the susceptibility difference per unit hematocrit between fully deoxygenated and fully oxygenated blood (typically around 1.8 × 10^−7^ in CGS units), and Hct represents the hematocrit level (percentage of red blood cells in whole blood). Actual Hct measurements from patients may be used, or established literature values may be applied, depending on the study design. In our study, we did not measure the Hct individually for each participant. This decision was based on the low prevalence of anemia in the population with OSA. Consequently, we relied on a previously established hematocrit value of 0.45 from the literature (Haacke et al. 2010). *P*_v_ is the partial volume effect correction factor that influences the accuracy of the measurements. *P*_v_ can affect the signal‐to‐noise ratio (CNR) depending on the vessel size. To account for this, we consulted a prior study that suggested a *P*_v_ correction factor of approximately 6.0 for a specific voxel ratio and vessel diameter (Ebrahimi et al. 2021).

To generate OEF maps encompassing the entire brain volume, we employed a sliding window technique. Imagine a small window that systematically traverses the brain image. Within each window, a developed in‐house MATLAB code calculated the OEF. This process ultimately creates a map that visualizes OEF variations across the whole brain.

The generated OEF map was linearly registered to the SWI‐magnitude image using SPM12 (http://www.fil.ion.ucl.ac.uk/spm/software/spm12) and ITK‐SNAP (http://www.itksnap.org/) software. Segmentation of brain regions and structures was performed using the T1‐Multiatlas (Mori et al., 2016). Following the automated segmentation, two experienced radiologists meticulously reviewed and verified the results for each participant.

### 2.7 Neuropsychological Assessments

A battery of cognitive tests was performed in patients with OSA and healthy controls. Neuropsychological assessments included digit span (forward and backward) tests from the Wechsler Memory Scale‐Revised, Trail Making Tests A and B, and the Stroop test, which assessed working memory, attention, and executive function. The Beck Depression Inventory (BDI) was used to evaluate the emotional status.

### 2.8 Statistical Analysis

Continuous variables are expressed as the mean ± SD, whereas categorical variables are represented as the number of occurrences (*n* counts), along with the percentage (%). The Kolmogorov–Smirnov test was used to assess the normality of the data distribution. The independent samples *t*‐test or Mann–Whitney *U* test was used to compare continuous variables, whereas the *χ* ^2^ or Fisher exact tests were used for categorical variables. The findings of the correlation analysis were presented as Pearson or Spearman correlation coefficients. All statistical tests were two‐tailed, with a significance level of *p* < 0.05. Bonferroni correction for multiple testing was used to account for the increased risk of Type 2 errors when conducting multiple comparisons. SPSS Statistics software (IBM Corporation, v. 24) was used for all the analyses.

## 3 Results

### 3.1 Demographic and Clinical Characteristics

Regarding age, sex, BMI, education level, and dominant hand (all right‐handed), all participants in the control group were matched to those in the OSA group. Table 1 shows the participants' demographic and clinical characteristics, and Table 2 presents the PSG findings of the patients with OSA.

**TABLE 1:** Demographic and clinical characteristics of the participants.

	OSA (*N* = 15)^a^	HC (*N* = 16)^a^	*p* value^b^	Effect size (ES)	ES interpretation^c^, ^d^
Sex, male (%)	12 (80%)	13 (81.25%)	0.93	NA	NA
Age (years)	47.67 ± 10.16	45.81 ± 10.72	0.62	0.177	trivial
BMI (kg/m^2^)	29.63 ± 3.05	28.66 ± 5.68	0.55	0.210	small
Education (years)	14.27 ± 5.05	16 ± 4.74	0.54	0.109	small
Disease duration (month)	36.07 ± 22.09	NA	NA	NA	NA
STOP‐BANG (score)	5.22 ± 2.46	0.94 ± 0.77	2.88 × 10^−7^	0.973	large
ESS (score)	12.14 ± 6.11	4.06 ± 2.18	1.76 × 10^−7^	1.414	large

Abbreviations: BMI, body mass index; ESS, Epworth Sleepiness Scale; HC, healthy control; NA, not applicable; OSA, obstructive sleep apnea.

^a^ Data presented as mean ± SD for continuous variables or *n* counts (%) for categorical variables.

^b^ *p* < 0.05 was considered significant.

^c^ The effect size used was Cohen's *d* (cut off; trivial: 0–0.19; small: 0.2–0.49; medium: 0.5–0.79; large: ≥ 0.8).

^d^ The effect size used was *r* (cut off; trivial: 0–0.09; small: 0.1–0.29; medium: 0.3–0.49; large: ≥ 0.5).

**TABLE 2:** PSG findings of the OSA patients.

AHI (number/h)	57.96 ± 38.17
ODI (number/h)	49.02 ± 28.87
SI (number/h)	304.81 ± 217.29
AI (number/h)	35.73 ± 32.65
REM Latency (min)	158.41 ± 99.86
Minimum SpO_2_ (%)	74.80 ± 9.02
Mean SpO_2_ (%)	91.53 ± 1.95
N1 (%)	25.81 ± 20.85
N2 (%)	55.72 ± 16.90
N3 (%)	9.08 ± 7.61
REM (%)	9.45 ± 5.45

*Note*: Data presented as mean ± SD.

Abbreviations: AHI, apnea–hypopnea index; AI, arousal index; ODI, oxygen desaturation index; OSA, obstructive sleep apnea; REM, rapid eye movement; SI, snoring index; SpO_2_, oxygen saturation.

### 3.2 OEF Findings

**FIGURE 2:**
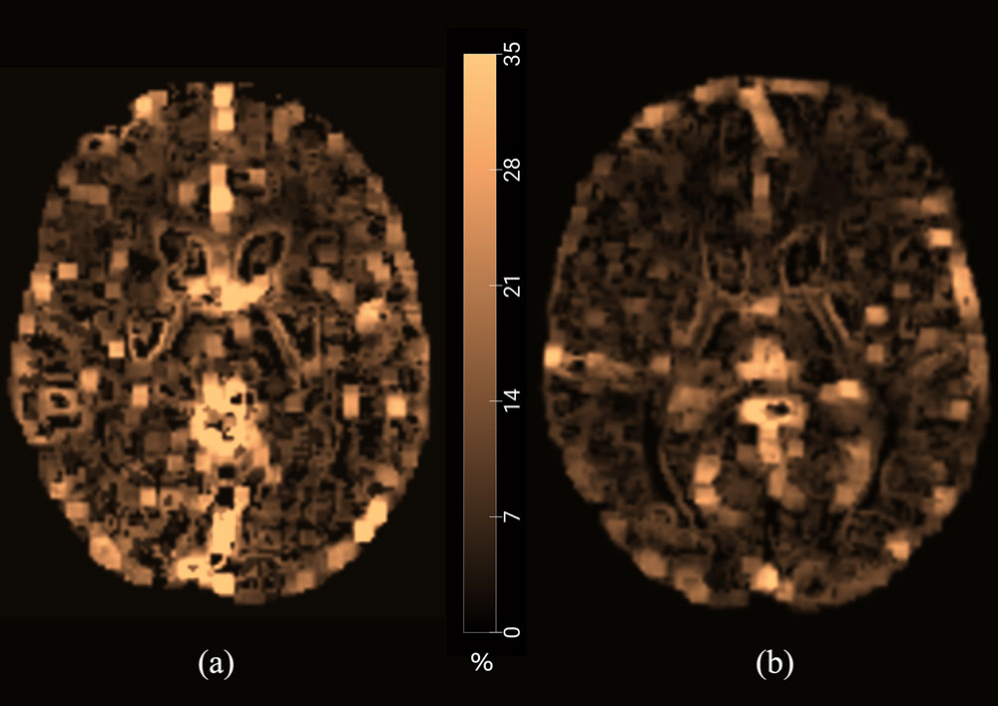
Generated OEF color map. (a) Healthy controls and (b) OSA patients.

The OEF map of one patient and one healthy control group is shown as an example in Figure 2. Table 3 shows the findings of the analysis, which involved segmenting 15 distinct regions in the output map and comparing the average values of each region between the two groups. The OEF values in patients with OSA were significantly lower than in healthy controls across all cerebral regions studied, but comparable in the cerebellar regions. In all cognitive functions, OSA patients showed a weaker performance than healthy controls. These differences were significant in backward digit span score (*p* = 0.005), backward digit span longest train length (*p* = 0.008), trail‐making B time (*p* = 0.040), and Stroop A time (*p* =  0.008).

**TABLE 3:** Comparison of OEF values and neuropsychological test results in OSA and healthy control.

	OSA (*N* = 15)^a^	HC (*N* = 16)^a^	*p* value^b^	Effect size (ES)	ES interpretation^c^, ^d^
OEF (%)					
Right hemisphere	46.99 ± 6.90	54.20 ± 7.35	0.009	1.010	Large
Left hemisphere	46.85 ± 6.89	54.41 ± 7.51	0.007	1.046	Large
Right cerebral cortex	29.15 ± 4.49	33.64 ± 5.53	0.020	0.886	Large
Left cerebral cortex	29.42 ± 4.49	34.20 ± 5.61	0.014	0.937	Large
Right frontal	40.70 ± 5.91	47.05 ± 7.65	0.016	0.923	Large
Left frontal	42.22 ± 6.74	49.75 ± 6.62	0.004	1.126	Large
Right parietal	41.26 ± 7.51	48.80 ± 8.15	0.012	0.961	Large
Left parietal	41.28 ± 7.35	48.38 ± 7.30	0.012	0.968	Large
Right temporal	48.97 ± 6.36	57.71 ± 8.43	0.003	1.164	Large
Left temporal	47.21 ± 6.58	55.14 ± 8.55	0.007	1.034	Large
Right occipital	54.48 ± 11.10	63.66 ± 7.83	0.012	0.961	Large
Left occipital	51.32 ± 8.55	59.24 ± 9.37	0.020	1.614	Large
Cerebellum	44.15 ± 6.60	48.69 ± 8.45	0.097	0.298	Small
Right cerebellum	44.41 ± 6.72	48.06 ± 9.67	0.235	0.436	Small
Left cerebellum	43.71 ± 6.80	49.09 ± 7.74	0.053	0.347	Medium
Neuropsychological test results					
Forward digit span score	7.53 ± 2.77	9.5 ± 2.40	0.055	0.356	Medium
Forward digit span longest train length	5.60 ± 1.54	6.35 ± 1.54	0.181	0.248	Small
Backward digit span score	4.86 ± 1.88	7.21 ± 2.15	0.005	0.519	Large
Backward digit span longest train length	3.80 ± 1.08	5.28 ± 1.48	0.008	0.493	Medium
Trail making A time (s)	28.47 ± 12.95	22.72 ± 6.07	0.407	0.153	Small
Trail making B time (s)	65.91 ± 23.07	48.49 ± 12.92	0.040	0.380	Medium
Stroop A time (s)	147.96 ± 53.93	111.23 ± 23.21	0.008	0.494	Medium
Stroop A errors	1.26 ± 1.62	0.71 ± 1.97	0.067	0.339	Medium
Stroop B time (s)	109.34 ± 39.57	89.38 ± 14.85	0.116	0.291	Small
Stroop B errors	0.33 ± 0.81	< 0.01 ± < 0.01	0.083	0.322	Medium
Stroop C time (s)	232.91 ± 83.99	182.31 ± 37.97	0.076	0.335	Medium
Stroop C errors	7.60 ± 8.46	2.92 ± 3.27	0.130	0.286	Small
Forward digit span score	7.53 ± 2.77	9.5 ± 2.40	0.055	0.356	Medium

^a^ Data presented as mean ± SD.

^b^ *p* < 0.05 was considered significant.

^c^ The effect size used was Cohen's *d* (cut off; trivial: 0–0.19; small: 0.2–0.49; medium: 0.5–0.79; large: ≥ 0.8).

^d^ The effect size used was *r* (cut off; trivial: 0–0.09; small: 0.1–0.29; medium: 0.3–0.49; large: ≥ 0.5).

In the assessment of patients with OSA, significant correlations were found between cerebral cortex OEF and N1/N2, as well as between frontal, temporal, and occipital OEF and N2, as listed in Table 4.

**TABLE 4:** Significant correlations between PSG outputs and OEF values.

	Correlation coefficient (*r*)	*p* value^*^
Right cerebral cortex OEF and N1	0.518	0.048
Right cerebral cortex OEF and N2	−0.680	0.005
Left cerebral cortex OEF and N2	−0.643	0.010
Right frontal OEF and N2	−0.527	0.044
Left frontal OEF and N2	−0.605	0.017
Right temporal OEF and N2	−0.681	0.005
Left temporal OEF and N1	0.521	0.047
Left temporal OEF and N2	−0.587	0.022
Right occipital OEF and N2	−0.596	0.019

* *p* < 0.05 was considered significant.

Correlation analyses revealed significant relationships between neuropsychological test performance and the mean OEF values across various brain regions in patients with OSA. The backward digit span score and longest train length were negatively correlated with OEF in the left cerebral cortex, left hemisphere, and frontal, parietal, temporal, and occipital regions. The correlation coefficients and *p* values for these analyses are presented in Table 5.

**TABLE 5:** Significant correlations between neuropsychological test results and OEF values in patients with OSA.

Neuropsychological test	Region	Correlation coefficient (*r*)	*p* value^*^
Backward digit span score	Left cerebral cortex	−0.597	0.019
	Left hemisphere	−0.610	0.016
	Right frontal	−0.549	0.034
	Left frontal	−0.621	0.014
	Left parietal	−0.653	0.008
	Left temporal	−0.518	0.048
	Left occipital	−0.616	0.015
Backward digit span longest train length	Right cerebral cortex	−0.629	0.012
	Left cerebral cortex	−0.703	0.003
	Right cerebellum	−0.561	0.030
	Left cerebellum	−0.559	0.030
	Right hemisphere	−0.606	0.017
	Left hemisphere	−0.651	0.009
	Cerebellum	−0.517	0.048
	Right frontal	−0.631	0.012
	Left frontal	−0.716	0.003
	Left parietal	−0.682	0.005
	Right temporal	−0.627	0.012
	Left temporal	−0.621	0.013
	Right occipital	−0.564	0.028
	Left occipital	−0.650	0.009

* *p* < 0.05 was considered significant.

### 3.3 QSM and Volumetry Findings

**FIGURE 3:**
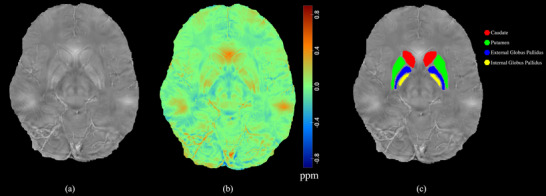
Reconstructed QSM map. (a) QSM in grayscale, (b) QSM color map, and (c) segmented caudate (red), putamen (green), external globus pallidus (blue), and internal globus pallidus (yellow) nuclei.

The reconstructed QSM map is shown in Figure 3. Following the segmentation of the map into 21 distinct regions, there were no significant differences between the OSA and healthy control groups in terms of quantitative susceptibility and volume, for each region.

**FIGURE 4:**
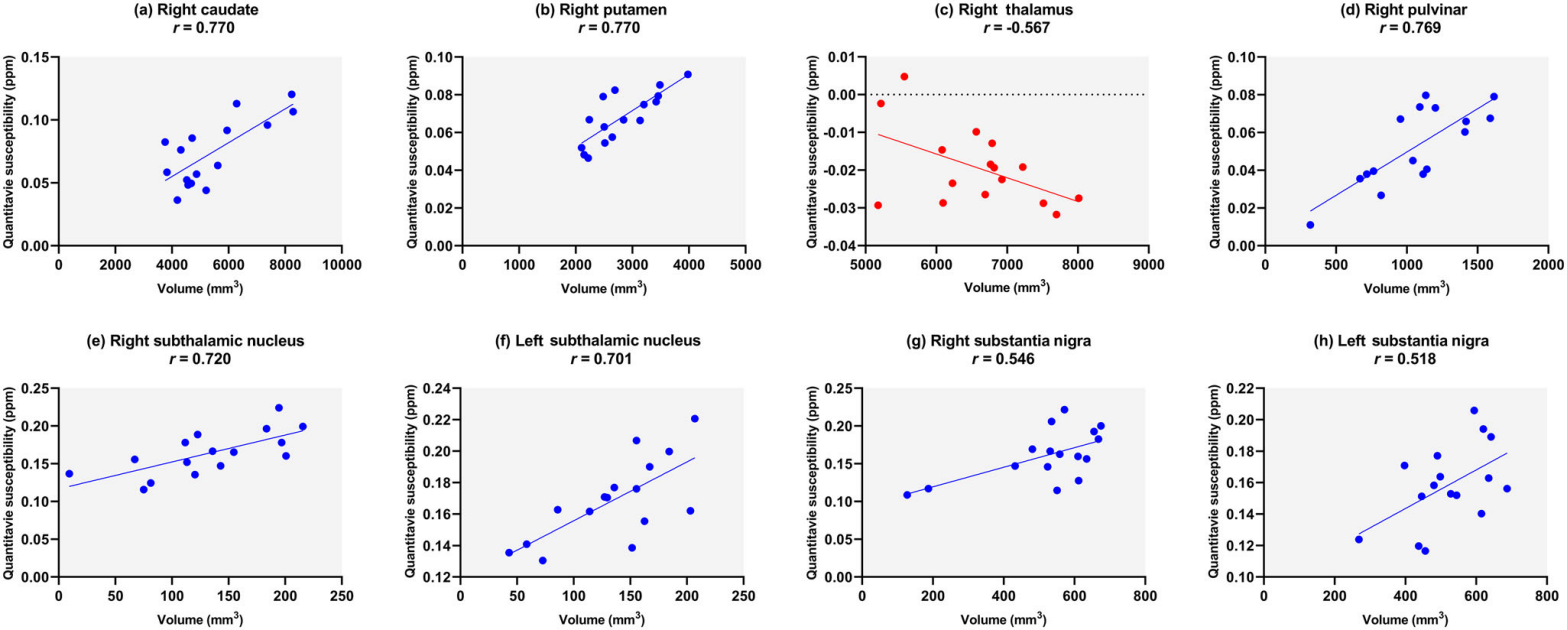
Significant correlations between quantitative susceptibility and brain volumes in OSA patients. (a) Right caudate, (b) right putamen, (c) right thalamus, (d) right pulvinar, (e) right subthalamic nucleus, (f) left subthalamic nucleus, (g) right substantia nigra, and (h) left substantia nigra.

Analysis of magnetic susceptibility and brain volume in patients with OSA and healthy controls revealed correlations between these measures in specific brain regions. The right caudate and right putamen had a greater positive correlation among the patients (*r* = 0.770, *p* = 0.001), whereas the right pulvinar had the highest negative correlation (*r* = −0.567, *p* = 0.027). In terms of the significant correlations found in both groups, in three out of five situations, the effect size was stronger in the OSA patients than in the healthy controls (right internal globus pallidus: 0.695 vs. 0.485, left red nucleus: 0.558 vs. 0.524, and right dentate: 0.570, 0.465). Figure 4 shows the brain regions where significant correlations between magnetic susceptibility and volume were observed exclusively in the OSA patient group.

Our analysis revealed significant correlations between specific brain regions identified by their quantitative susceptibility values and various sleep characteristics measured during PSG. Table 6 presents the details of these correlations. Notably, several subcortical structures in the left hemisphere, including the thalamus, subthalamic nucleus, and substantia nigra, correlated with sleep stages (N1, N3, and REM latency). Interestingly, both the left and right thalami correlated with snoring indices. Additionally, the table highlights correlations between specific brain regions (red nucleus, dentate nucleus) and sleep stages (REM, N1, N2) in both hemispheres.

**TABLE 6:** Significant correlations between PSG findings and quantitative susceptibility values.

	Correlation coefficient (*r*)	*p* value^*^
Left caudate and N1	−0.579	0.024
Right internal globus pallidus and REM	0.519	0.048
Right thalamus and snoring index	0.738	0.002
Right pulvinar and snoring index	0.646	0.009
Left subthalamic nucleus and N3	0.578	0.024
Left subthalamic nucleus and REM latency	−0.566	0.044
Left subthalamic nucleus and N1	−0.621	0.013
Left substantia nigra and REM latency	−0.566	0.044
Right red nucleus and REM	0.553	0.033
Left red nucleus and N3	0.527	0.043
Right dentate and N1	−0.596	0.019
Right dentate and N2	0.520	0.047
Left dentate and N1	−0.514	0.049
White matter and REM	−0.562	0.029

* *p* < 0.05.

## 4 Discussion

To the best of our knowledge, this study is the first to investigate the relationship between brain iron content (measured by QSM), oxygen extraction fraction (OEF), and cognitive function in OSA patients compared with healthy controls

The decreased OEF detected in patients with OSA offers evidence of compromised cerebral oxygen delivery and energy metabolism, reflecting the underlying vascular dysfunction. OEF has been established as a marker for evaluating the balance between cerebral blood flow and oxygen demand in tissues (Jiang and Lu 2022). OEF measured by the QSM offers advantages over traditional methods, such as PET. QSM is noninvasive, avoids radiation exposure, and provides a higher spatial resolution (Kudo et al. 2016). A significantly lower OEF in OSA patients than in controls suggests reduced oxygen extraction in the brain, potentially due to microvascular dysfunction or impaired oxygen metabolism. This aligns with previous research demonstrating that OSA can lead to cerebral hypometabolism (Mubashir et al. 2019). The reason for the decrease in OSA patients' OEF can be one of the factors or a combination of these: reduced oxygen metabolism, increased blood flow, and potential for insufficient extraction. Some studies have shown that patients with OSA have a lower cerebral metabolic rate of oxygen (CMRO_2_), indicating that the brain might use less oxygen due to inefficiency or adaptation (Jensen et al. 2018). Wu et al. (2022) found lower baseline CMRO_2_ in OSA subjects compared to healthy controls during normal breathing. The body's initial response to low oxygen levels is to increase the blood flow to deliver more oxygen to the brain. This might lead to a relatively smaller difference between the arterial and jugular venous oxygen content, which contributes to the calculation of OEF. Despite the increased blood flow, studies suggest that OEF might not be enough to compensate for oxygen deprivation during apneas, leading to a net reduction in OEF (Bradley and Floras 2009).

The positive correlation between the right cortical OEF and time spent in N1 sleep suggests that higher oxygen demand in this region may disrupt the transition to deeper sleep (Yan et al. 2021). Negative correlations were observed between frontal, temporal, occipital, and cerebral cortical OEF and N2 sleep. As N2 is a deeper non‐REM stage, this implies that a higher regional OEF impairs consolidation into deeper sleep (Joosten et al. 2021). From another point of view, it is also possible that the increased OEF reflects localized hypoxic stress that influences sleep architecture in these regions.

The cognitive impairment observed across domains in patients with OSA is consistent with the findings of previous studies. Multiple studies have demonstrated deficits affecting executive functions, verbal and visual memory, and aspects of attention processing (Andrade et al. 2018; Bucks et al. 2017). The significant differences seen here on neuropsychological tests sensitive to functions such as digit span, trail making, and Stroop tests provide further validation. The findings suggest that changes in how oxygen is delivered to different parts of the brain during disrupted sleep may contribute to cognitive problems over time, affecting some brain functions more than others (Yan et al. 2021; Dang‐Vu et al. 2010). Notably, higher OEF across the frontal, parietal, temporal, and occipital cortices correlates negatively with backward digit span performance, implicating vascular dysfunction reflected by OEF in working memory networks vulnerable to sleep disruption (Okuda et al. 2021).

A previous study had shown that at the hemisphere level, there was a strong correlation between QSM‐OEF and PET‐OEF (Kudo et al. 2016). However, QSM is already known to be among the most accurate MRI‐based techniques for determining OEF (Jiang and Lu 2022). For QSM analysis, improving the source image's spatial resolution is extremely valuable as it offers more specific information on the location and shape of veins. Since veins play a crucial role in oxygen extraction, a clearer picture of their size and distribution can significantly improve the accuracy of local OEF measurements (McFadden et al. 2021). In this study, by acquiring high‐resolution data, the accuracy of measurements likely increased due to the improved ability to differentiate between tissues and small blood vessels. While PET offers valuable information on OEF, QSM‐based MRI methods hold promise as a replacement due to their inherent advantages. MRI offers a noninvasive, nonionizing approach, making it safer and more accessible for repeated studies in clinical populations. However, the development of PET/MRI scanner technology allows for the possibility of combining these methods in future researches. This could be particularly valuable for comparative studies and potentially for further improving the accuracy of OEF measurements by leveraging the strengths of both techniques.

This may be due to the fact that calculating local OEF depends on the degree of partial volume effects and segmentation of veins as well as the concentration of deoxygenated hemoglobin. Therefore, improving the quality or resolution of the source image for QSM analysis to provide more detail on the presence and geometry of veins would likely increase the accuracy of local OEF measurement. In addition, optimizing scanning parameters and QSM postprocessing could also lead to more precise quantification of OEF.

The QSM was not significantly different between patients with OSA and healthy controls. Increased QSM observed regionally has been shown in other conditions involving iron dyshomeostasis, such as Parkinson's disease (Alushaj et al. 2023; Sun et al. 2020). One study found QSM could detect substantia nigra iron accumulation in idiopathic REM sleep behavior disorder (iRBD), proposed to mark a prodromal α‐synucleinopathy stage (Sun et al. 2020). Abnormal iron metabolism has been implicated in other sleep disorders. Another study observed thalamic, caudate, and pulvinar changes following RLS iron treatment, linking regional deficiencies to symptoms (Kim et al. 2023). Our findings suggest that there may not be a significant correlation between OSA and iron deposition; however, the positive correlation between the volume and magnetic susceptibility of the left and right substantia nigra should be considered as a potential consequence.

The region‐specific correlations reported between QSM values and various PSG measures provide initial insights into how disrupted sleep may differentially affect regional iron homeostasis. The lack of evidence makes it challenging to demonstrate with certainty whether changes in PSG measurements lead to iron deposits in specific brain regions or vice versa. However, based on this study, which revealed a positive correlation between sleep duration and oxygen deposition in deep sleep stages (N3) and a negative correlation between sleep duration and iron deposition in lighter stages (N1), it can be concluded that the probability of iron deposition is lower in lighter stages. This data also aligns with the sleeping patterns of OSA patients, who typically experience longer times of light sleep (Kerkamm et al. 2023; Shahveisi et al. 2018). Consequently, these findings appear to be in line with our previous findings, which showed that the brains of patients with OSA did not exhibit significant iron accumulation.

The SI showed a positive correlation with thalamus QSM. The generation and preservation of the sleep–wake cycle are functions of the thalamus, in addition to other brain areas such as the brainstem and hypothalamus (Gent, Bassetti, and Adamantidis 2018; Jan et al. 2009). Research has demonstrated that the severity of sleep disturbance and sleep‐disordered breathing could be correlated with alterations in thalamic volume, metabolic activity, and functional connectivity (Gilman et al. 2003; Motomura et al. 2021; Santarnecchi et al. 2022). Control of respiratory function, which is closely connected to sleep‐related breathing disorders, is another function of the thalamus. The hypothalamus has many nuclei involved in respiratory control, modulating respiration under hypoxic and hypercapnic conditions, awake and sleep states, and under stress. Hypothalamic dysfunction can cause abnormal breathing and hypoventilation (Fukushi, Yokota, and Okada 2019). Therefore, the right thalamus is linked to snoring severity, suggesting that brain changes affect sleep‐breathing.

This was the first study to evaluate magnetic susceptibility (QSM) and OEF as potential contributors to cognitive decline in this population. However, this study had several limitations. First, the study had a small sample size, which limited the generalizability of the findings. Second, the inclusion of the control group without a PSG study, relying solely on clinical examination and a STOP‐BANG questionnaire, may have included individuals with undetected moderate sleep apnea. Third, it is important to interpret the QSM results cautiously because the iron level in the brain does not affect the QSM value by itself. Diamagnetic myelination and finely oriented white matter may contribute to magnetic susceptibility, which could complicate interpretation, even though iron is the primary source of susceptibility, as determined by QSM. Larger prospective and longitudinal investigations are needed to validate whether QSM and OEF can truly serve as clinically meaningful biomarkers for disease monitoring, severity tracking, and treatment response evaluation in OSA.

## 5 Conclusions

This study provides novel insights into the neurophysiological correlates of cognitive impairment in OSA patients. The findings revealed a clear association between alterations in the regional brain OEF and performance on neuropsychological tests. These results suggest that disruptions in cerebral oxygen metabolism, rather than structural brain changes, may be a more prominent feature underlying cognitive deficits experienced by individuals with OSA. Identification of the OEF as a potential biomarker for the neural mechanisms linking sleep‐disordered breathing to cognitive impairment opens new avenues for further research and the development of targeted interventions to address the neurological consequences of this prevalent sleep disorder.

## Author Contributions

**Mahdi Mohammadi**: conceptualization, methodology, software, data curation, writing–original draft, investigation, visualization, validation, formal analysis, writing–review and editing. **Shahram Samadi**: methodology, supervision, validation, formal analysis, writing–review and editing, conceptualization. **Seyed Amir Hossein Batouli**: conceptualization, methodology, software, validation, investigation, formal analysis, writing–review and editing. **Khalil Pestei**: writing–review and editing, conceptualization. **Mohammad Ali Oghabian**: conceptualization, methodology, supervision, validation, formal analysis, writing–review and editing; project administration.

## Ethics Statement

Written consent was obtained from all the participants with potentially identifiable images or data. The study was approved by the Ethics Committee of the Tehran University of Medical Sciences.

## Conflicts of Interest

The authors declare no conflicts of interest.

### Peer Review

The peer review history for this article is available at https://publons.com/publon/10.1002/brb3.70273.

## Data Availability

This article contains all the data produced or analyzed during this investigation. Further inquiries should be forwarded to the corresponding author.
